# The biomechanical analysis of three plating fixation systems for periprosthetic femoral fracture near the tip of a total hip arthroplasty

**DOI:** 10.1186/1749-799X-5-45

**Published:** 2010-07-23

**Authors:** James P Lever, Rad Zdero, Markku T Nousiainen, James P Waddell, Emil H Schemitsch

**Affiliations:** 1 Peterborough Regional Health Centre, 204A - 849 Alexander Court, Peterborough, ON, K9J-7H8, Canada; 2 Martin Orthopaedic Biomechanics Laboratory, Shuter Wing (Room 5-066), St. Michael's Hospital, 30 Bond Street, Toronto, ON, M5B-1W8, Canada; 3 Department of Mechanical and Industrial Engineering, 350 Victoria St., Ryerson University, Toronto, ON, M5B-2K3, Canada; 4 Division of Orthopaedic Surgery, St. Michael's Hospital, Manulife Building, 1002A - 2 Queen Street East, Toronto, ON, M5C-3G7, Canada; 5 Division of Orthopaedic Surgery, St. Michael's Hospital, 800 - 55 Queen Street East, Toronto, ON, M5C-1R6, Canada

## Abstract

**Background:**

A variety of techniques are available for fixation of femoral shaft fractures following total hip arthroplasty. The optimal surgical repair method still remains a point of controversy in the literature. However, few studies have quantified the performance of such repair constructs. This study biomechanically examined 3 different screw-plate and cable-plate systems for fixation of periprosthetic femoral fractures near the tip of a total hip arthroplasty.

**Methods:**

Twelve pairs of human cadaveric femurs were utilized. Each left femur was prepared for the cemented insertion of the femoral component of a total hip implant. Femoral fractures were created in the femurs and subsequently repaired with Construct A (Zimmer Cable Ready System), Construct B (AO Cable-Plate System), or Construct C (Dall-Miles Cable Grip System). Right femora served as matched intact controls. Axial, torsional, and four-point bending tests were performed to obtain stiffness values.

**Results:**

All repair systems showed 3.08 to 5.33 times greater axial stiffness over intact control specimens. Four-point normalized bending (0.69 to 0.85) and normalized torsional (0.55 to 0.69) stiffnesses were lower than intact controls for most comparisons. Screw-plates provided either greater or equal stiffness compared to cable-plates in almost all cases. There were no statistical differences between plating systems A, B, or C when compared to each other (p > 0.05).

**Conclusions:**

Screw-plate systems provide more optimal mechanical stability than cable-plate systems for periprosthetic femur fractures near the tip of a total hip arthroplasty.

## Background

Although uncommon, femoral fractures do occur in approximately 0.1% to 6% of all total hip arthroplasty cases [1-4]. These are most often found in either osteopenic elderly women or in patients who have experienced loosening of the femoral component [1,5-8].

Several factors likely predispose patients to periprosthetic femur fracture [6], including cracks or defects generated intra-operatively, regions in the bone that are not bypassed with a sufficiently long stem, prior hip surgery, and cortical thinning caused by a loose femoral component. Moreover, the tip of the hip stem itself functions as a stress riser and is one of the contributing factors in such fractures.

Periprosthetic femoral fractures may be categorized according to their occurrence in the proximal, middle, or distal areas of the femur [9]. Proximal fractures usually involve longitudinal splits that occur intra-operatively which may require specific interventions if unstable. Middle-region fractures occur between the lesser trochanter and the prosthetic tip, are linked with prosthetic loosening, and often appear post-operatively. Distal fractures occur either in the post-operative period below well-fixed components or intra-operatively when an uncemented femoral stem impacts the intra-medullary wall anteriorly.

Femoral fractures at the tip of a total hip arthroplasty stem have been classified as Vancouver B1 fractures [10,11]. These are known to be the most complex to manage, have been reported to comprise as many as 75% of all periprosthetic fracture cases, are associated with the most complications (such as non-union in 25 to 42% of all cases treated non-operatively), and are still a point of controversy as to which surgical intervention is best [1,5,12-15]. However, it should be noted that Type B2 fractures (i.e. hip implant is loose) and especially Type B3 fractures (i.e. hip implant is loose accompanied by substantial loss of bone stock) quite often necessitate a more complex reconstruction of the proximal femur than that required for Type B1 fractures (i.e. hip implant is stable).

The goals of treatment for a periprosthetic femur fracture at the tip of a femoral stem include successful fracture union while maintaining longterm implant survival. The most common approach is some variation of the Ogden-type construct, which involves placement of a metal plate laterally on the femur, proximal fixation using cables, and distal fixation with bicortical screws [16]. Other similar techniques incorporate various combinations of allograft struts, plates, and cerclage wires [9]. Although these approaches are used clinically, relatively few studies have been undertaken to quantitatively determine the biomechanical stability of these periprosthetic fracture constructs [1,4,17-24]. Moreover, previous investigations have examined the use of proximal cables or screws with plate fixation or have compared constructs using plate fixation and allograft struts. No studies, however, have directly compared cable-plate systems where the method of cable capture by the plate varied between the repair systems. Furthermore, these studies have limited their mechanical tests to standard axial, lateral, and torsional orientations, without considering clinically relevant four-point antero-posterior bending tests, four-point medio-lateral bending tests, and tests with the hip in flexion.

Therefore, the present purpose was to assess the biomechanical performance immediately following surgery of 3 cable-plate and screw-plate fixation systems used to repair periprosthetic femur fractures near the tip of a total hip arthroplasty. It was hypothesized that screw-plate versus cable-plate systems would yield higher biomechanical stiffnesses compared to intact control femurs. The clinical and biomechanical relevance of this study lies in the fact that the optimal solution remains elusive for this injury pattern. There is no "gold standard" technique for B1 periprosthetic fractures that has been widely accepted by investigators. Thus, there is a need for more reports on the mechanical properties of a variety of repair constructs to appear in the literature for the B1 fracture pattern.

## Methods

### Femur Specimens

Twelve pairs of fresh-frozen cadaveric femora were harvested from anonymous human donors. The specimens were wrapped and frozen at -20 degrees C. All femora were radiographed prior to inclusion in the study and were reviewed independently by 2 investigators. Any paired femora with osteolytic lesions, significant osteopenia (Engh index < 4), or previous fracture were excluded. The study was approved by the authors' institutional research and ethics board.

### Insertion of Total Hip Arthroplasties and Creation of Femoral Osteotomies

Each left femur was prepared for the cemented insertion of the femoral component of a total hip implant. Identical hip stems were inserted in a neutral position in the medullary canals. Right femora served as matched intact controls, since management of periprosthetic femur fractures may be improved by using fixation systems with equal or improved stability compared to healthy intact bone. Moreover, anatomic symmetry is expected to reduce any variability involving mechanical bone properties, thereby increasing the chances of discovering statistical differences between specimens if they exist [25-27]. Although some have expressed concern that left and right femora are not necessarily equivalent [28], other reports indicate no differences between them [29]. Following insertion of the implant, the distal tip of the stem was located by measuring along the outside of the femur from the top of the hip implant. An oscillating saw was then used to create a 45-degree oblique osteotomy at this level to represent a Vancouver B1 periprosthetic femur fracture [10,11]. The osteotomies were provisionally stabilized with bone forceps prior to definitive fixation.

### Application of Constructs for Femur Fracture Fixation

**Figure 1:**
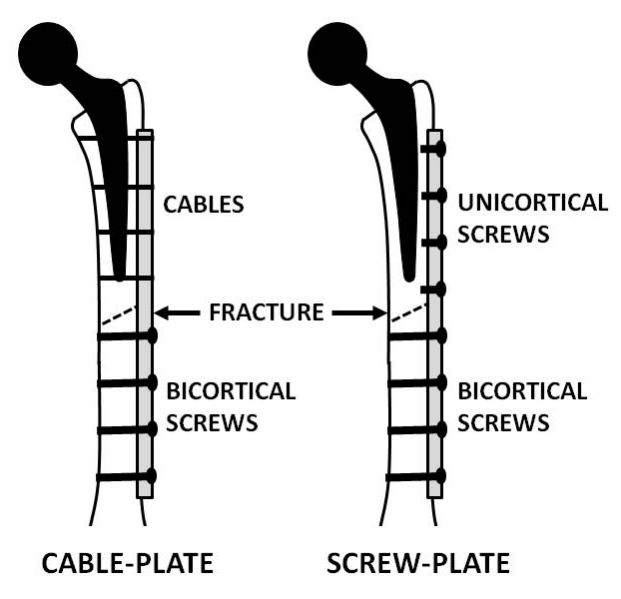
**Fracture repair constructs**. Each of the 3 cable-plate systems (constructs A, B, and C) were mechanically tested and subsequently transformed into 3 screw-plate systems for retesting. For the cable-plate setup, construct A and C had 4 single-cable loops applied proximally as shown, whereas construct B had double-wire loops at each of the 4 proximal locations.

Left femurs were randomly assigned to 3 groups to receive fracture fixation devices (Figure 1). All cable-plate systems were located on the lateral aspect of the femur and centered over the osteotomy. The construct systems created were as follows.

*Construct A *was the Zimmer Cable Ready System (Zimmer, Warsaw, IN, USA). It was designed to incorporate the cable into the plate. Four 316L stainless steel 1.8-mm cables were used in the study along with the 8-hole 246-mm plate. Each cable was passed through the plate via 2 transverse tunnels situated in between the plate holes. The cable was then tightened with a custom tensioner and locked by turning a set screw within the plate. Distal to the osteotomy, 4 cortical screws of 4.5-mm diameter were used to provide bicortical fixation using standard plating techniques.

*Construct B *was the AO Cable-Plate System (Synthes, Paoli, PA, USA). It was comprised of a broad 4.5-mm 14-hole dynamic compression plate of 250 mm length with 316L stainless steel wire mounts and 316L stainless steel double Luque cerclage wires. This construct utilized individual wire mounts inserted into the screw holes to provide a means for stable wire fixation. The wire mounts were inserted from the underside of the plate holes, such that the mounts protruded beyond the top surface of the plate to provide access for the Luque wire. A double Luque wire was inserted into 4 proximal wire mounts and then tensioned by manual twisting. Bicortical screw fixation was used to secure the plate distally, as described earlier.

*Construct C *was the Dall-Miles Cable Grip System (Howmedica, Rutherford, NJ, USA). It involved the application of a Dall-Miles stainless steel 316L plate with an alternating hole-and-slot design. The slots accepted cable sleeves that were inserted onto the outer surface of the plate, which was a 9-hole 254-mm plate with 10 cable slots. Each of 4 cables of 1.8-mm diameter was passed through a cable sleeve and around the bone to provide stable fixation. A custom tensioner was used to tighten the cables. The cable sleeve was crimped to provide capture. Distal bicortical screw fixation remained constant with 4 stainless steel cortical screws of 4.5-mm diameter.

Surgical stainless steel (grade 316L) is an austenite iron alloy used specifically for medical devices such as screws, cables, and wires. The iron matrix of 316L typically contains chromium (16-18%), nickel (10-14%), molybdenum (2-3%), and carbon (< 0.03%), depending on the application. Chromium improves scratch and corrosion resistance. Nickel provides a polished smooth surface. Molybdenum increases hardness. Carbon provides strength.

Following the testing of the 3 cable-plate systems, the cables proximal to the osteotomy were removed and replaced with 4 unicortical screws to create 3 screw-plate systems. The mechanical tests were repeated.

### Mechanical Testing

Each specimen was thawed at room temperature prior to testing. The distal condyles were removed such that the femurs were of the same working length. The distal femoral shafts were then potted with methyl-methacrylate in steel tubes (5 cm diameter × 10 cm length) so that the shafts were flush with the bottom of the steel tubes. The potted specimens were mounted and secured into a custom jig that could be adapted to orient the femoral shaft in abduction and forward flexion. An acetabular component was fixed to a load cell. Deforming forces were then applied to test the biomechanical stiffness of the constructs. An Instron 8501 machine (Instron, Norwood, MA, USA) was used for all mechanical testing. Saline solution was applied to the specimens during testing in order to minimize any mechanical changes that could be caused by bone drying.

**Figure 2:**
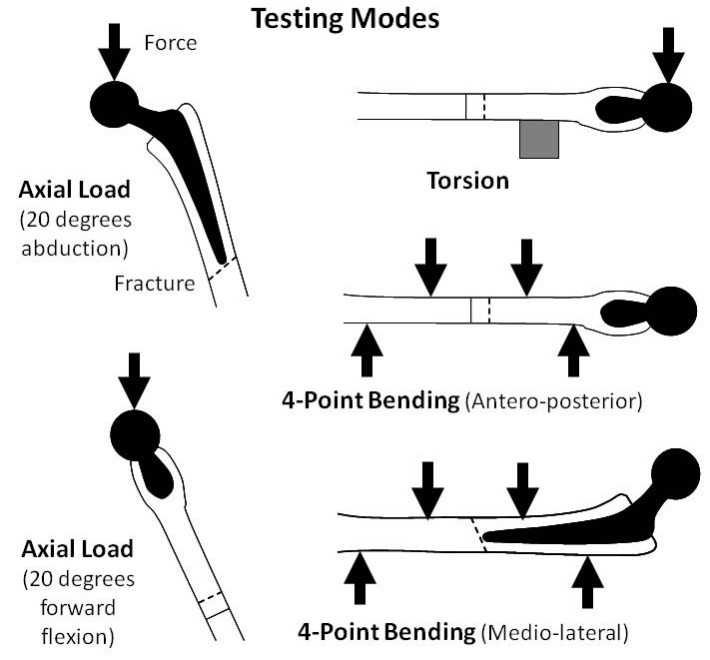
**Mechanical test modes**. Axial compression, torsion, and four-point bending.

Five mechanical test modes were used, namely axial compression (2 types), torsion, and four-point bending (2 types) (Figure 2). Thus, the total number of test cases was 5 test modes × 3 construct types = 15. For axial compression tests, a vertical force of 250 N was applied to the femoral head. The femurs were tested in 2 separate orientations, namely at 20 degrees of abduction and 20 degrees of forward flexion in order to produce shear on the construct, rather than trying to simulate single-legged stance as done by some investigators. For torsional tests, the femurs were oriented horizontally to simulate 90 degrees of flexion, and a vertical 250 N force was applied onto the anterior aspect of the femoral head to produce internal rotation. A support was placed distal to the intertrochanteric region to minimize long-axis bending. No computational corrections were required to account for variable neck length of the specimens since matched contralateral femurs acted as intact controls. Regarding four-point bending tests, antero-posterior and medio-lateral forces of 250 N perpendicular to the shaft were applied by indenters that were located symmetrically on either side of the osteotomy site. For each test mode, the slope of the load-versus-deflection curve was used to compute the stiffness of each test run. Each test run was repeated 4 times to obtain an average for a given testing mode.

Load levels of 250 N applied presently are far below that experienced physiologically at the proximal femur. These loads were chosen for several reasons. Firstly, the nature of the study was comparative, in that the relative performance between construct groups in the lab would be expected to translate to the "real-world" clinical situation, i.e., if used clinically in vivo under identical physiological and mechanical conditions, the ratios of the mechanical stiffnesses between the different constructs would be similar to that reported presently. Secondly, low loads ensured that the specimens remained within the linear elastic region of their load-versus-displacement behaviour, thereby eliminating any permanent deformation of the femurs so that all testing could be completed. Thirdly, previous studies in the literature acted as a precedent for similar load levels and/or regimes [1,4,17-24].

### Statistical Analysis

Stiffness data (left femurs) were expressed as a percentage of baseline of intact stiffness (right femurs) and were used to detect the relative effect of construct configuration on stiffness. One-way analyses of variance (ANOVA) were performed on the data with a significance level of 0.05 to determine the effect of construct on biomechanical behaviour. If warranted, post hoc multiple comparisons were made with unpaired student's t-tests between groups.

## Results

### Axial Stiffness

**Figure 3:**
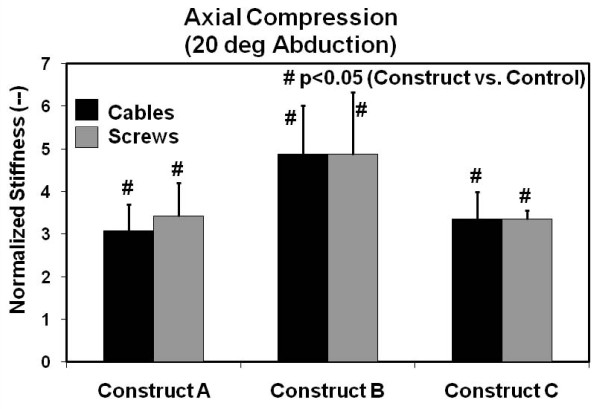
**Axial stiffness results for 20 degrees of abduction**. Values are normalized with respect to the intact right femur control group. Error bars are the standard errors of the mean.

Axial compression test results at 20 degrees of abduction are shown in Figure 3. There was a statistically significant increase in normalized stiffness (average = 3.82, range = 3.08 to 4.88) (p < 0.038) over intact control specimens for both cable-plate and screw-plate systems for all constructs considered. Cable-plate and screw-plate systems were equally stiff for a given construct A, B, or C (p > 0.05) and within this test mode when all systems were combined as a single group (p = 0.308).

**Figure 4:**
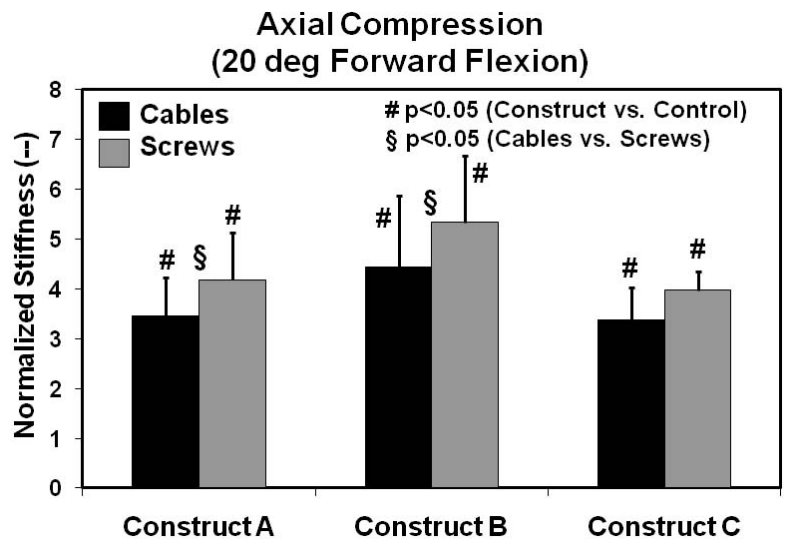
**Axial stiffness results for 20 degrees of forward flexion**. Values are normalized with respect to the intact right femur control group. Error bars are the standard errors of the mean.

Axial compression test results at 20 degrees of forward flexion are provided in Figure 4. There was a statistically significant improvement in normalized stiffness (average = 4.12, range = 3.38 to 5.33) (p < 0.047) over intact control specimens for both cable-plate and screw-plate systems for all constructs considered. Screw-plate systems were stiffer than cable-plate systems for Constructs A and B (p < 0.046), but they were equally stiff for Construct C (p = 0.23). When all constructs were combined into one group for this test mode, screws provided improved stiffness over cables (p = 0.007).

Finally, there was no statistical difference between the plating Constructs A, B, or C, when either proximal screws or cables were used (p > 0.05), for both axial test modes.

### Torsional Stiffness

**Figure 5:**
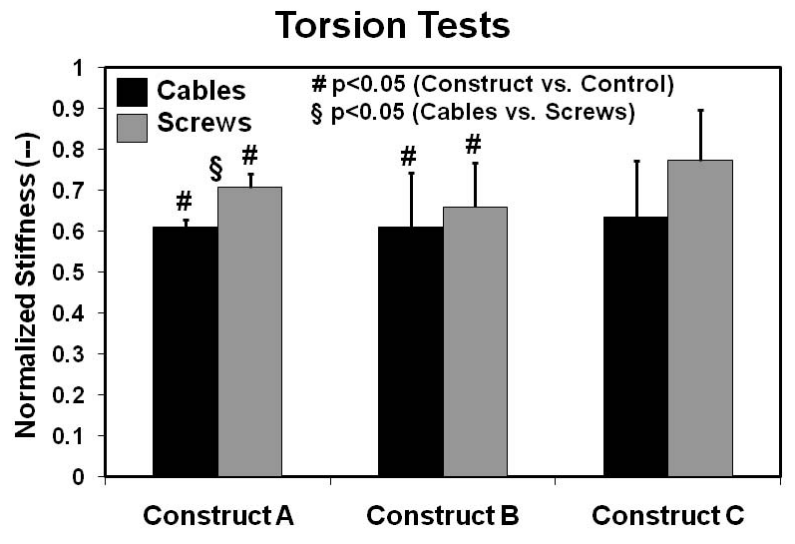
**Torsional stiffness results**. Values are normalized with respect to the intact right femur control group. Error bars are the standard errors of the mean.

Torsion test results for internal rotation of the femoral head are shown in Figure 5. Cable-plate and screw-plate systems for Constructs A and B were much less stiff (average = 0.62, range = 0.55 to 0.69) (p < 0.029) compared to control femurs. Construct C, however, provided as much stiffness as intact control specimens for both cable-plate and screw-plate configurations (p > 0.05). In modifying a cable-plate to a screw-plate system, there was no improvement in stiffness for Constructs B and C (p > 0.086), but there was improvement for Construct A (p = 0.044). When constructs were combined together into one group for this test mode, screws provided greater stiffness than cables (p = 0.04). Lastly, there was no statistical difference between the plating Constructs A, B, or C for either proximal screws or cables (p > 0.05).

### Four-Point Bending Stiffness

**Figure 6:**
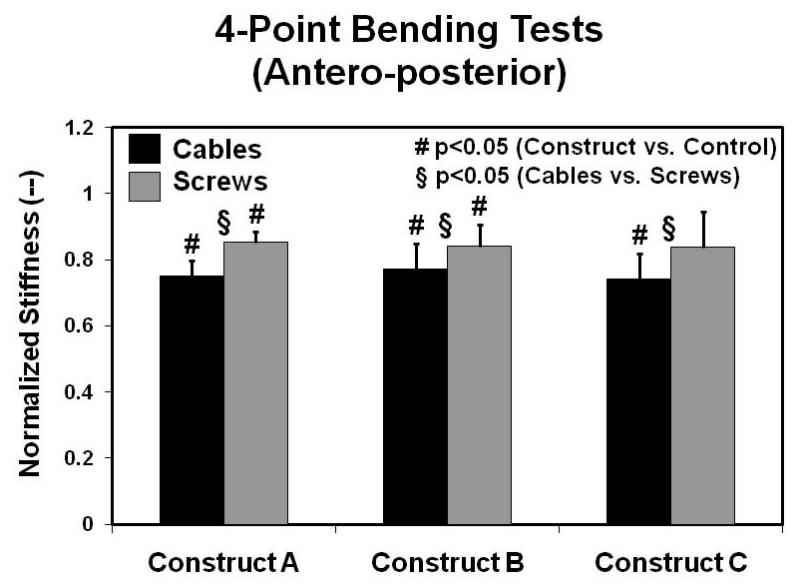
**Antero-posterior four-point bending stiffness results**. Values are normalized with respect to the intact right femur control group. Error bars are the standard errors of the mean.

Four-point bending results for normalized stiffness in the antero-posterior plane are given in Figure 6. There was a moderate drop in stiffness with respect to control values (average = 0.79, range = 0.74 to 0.85) (p < 0.045) for most of the cable-plate and screw-plate systems. The screw-plate configuration of Construct C, however, was able to maintain stiffness equal to that of the control specimen (p = 0.114). In modifying a cable-plate to a screw-plate system, there was a statistically significant improvement for a given construct (p < 0.04). When constructs were grouped together for this test mode, screws provided improved stiffness over cables (p < 0.05).

**Figure 7:**
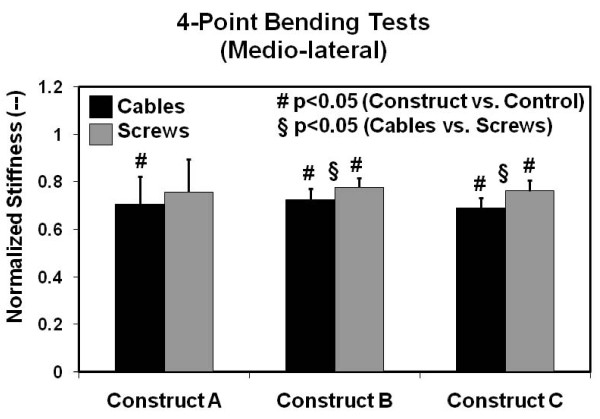
**Medio-lateral four-point bending stiffness results**. Values are normalized with respect to the intact right femur control group. Error bars are the standard errors of the mean.

Four-point bending results for normalized stiffness in the medio-lateral plane are illustrated in Figure 7. Significant reductions in stiffness from control (average = 0.73, range = 0.69 to 0.78) (p < 0.044) were noted for most of the systems tested. However, the screw-plate configuration of Construct A was as stiff as the control femur (p = 0.088). Screw-plate systems were stiffer than cable-plate systems for Constructs B and C (p < 0.006), but were not stiffer for Construct A (p = 0.07). When constructs were combined into one group for this test mode, screws provided improved stiffness over cables (p < 0.05).

For both four-point bending configurations, there were no statistical differences between any of the plating Constructs A, B, or C, when either proximal screws or cables were used (p > 0.05).

## Discussion

### General Findings

An optimal solution still remains elusive for repairing periprosthetic femur fractures near the tip of a total hip arthroplasty [1,5,12-15]. The aim at present, therefore, was to evaluate the biomechanical performance immediately following surgery of 3 cable-plate and screw-plate fixation systems. Previous studies have not directly compared different cable plating systems for periprosthetic femoral fracture fixation. Earlier investigations have examined proximal cables or screws with plate fixation or have compared constructs using plate fixation and allograft struts. This study, however, is the first investigation to directly compare 3 cable-plate systems where the method of capture of the cable by the plate varies between the 3 systems.

The management of periprosthetic femoral fractures may be improved by the use of fixation systems that provide equal or improved stability compared to healthy intact bone. At present, axial tests demonstrated a vast improvement in stiffness of 3.08 to 5.33 times that of intact controls. However, four-point normalized bending stiffnesses (0.69 to 0.85) and torsional normalized stiffnesses (0.55 to 0.69) were much lower than control femurs, except in a few instances. This implies that the fixation methods examined at present provide reasonable stability for patients who post-operatively engage in simple activities that place the femur in axial compression over a limited range of motion, rather than torsion or bending.

This investigation suggests that screw-plate systems may offer more optimal stability than cable-plate systems, when using a plate applied laterally on the femur. Recall that the total number of test cases was 5 test modes × 3 construct types = 15. For a given construct, screw-plate systems were either stiffer than their cable-plate counterparts (8 of 15 cases) or equally as stiff (7 of 15 cases). For a given mechanical testing mode, screw-plate systems were stiffer than cable-plate systems in 4 of 5 test modes and equally stiff in 1 of 5. A surgical advantage of screw fixation is that no circumferential tissue stripping is required, as in the case of cables or wires. Moreover, screw-plate systems have been shown to produce union rates of about 90 to 100% [9]. There are some concerns with screw fixation, however, namely that replacing proximal cables with proximal unicortical screws in the vicinity of a THA can create stress risers in local bone leading to refracture [9]. The increased strength afforded by cortical screws placed near (or through) the cement mantle is offset by the risk of prosthesis loosening due to violation of the cement mantle, although clinical evidence for this is lacking [9,13,30].

### Comparison to Prior Investigations

For stiffness values, this investigation yielded similar conclusions to that of previous studies that assessed the use of proximal unicortical screws in place of cables for Ogden-type constructs. Dennis and co-workers tested 4 lateral plate constructs and one allograft double-strut construct with various combinations of proximal and distal screws and cables [1]. Their tests included lateral bending, torsion, and axial compression with the femur in 25 degrees of adduction. They found that a construct with proximal unicortical screws and distal bicortical screws was the stiffest in lateral bending and second stiffest in torsion and axial compression, being surpassed only by a construct that proximally combined both screws and cables. Similarly, Schmotzer and colleagues concluded that the use of proximal unicortical screws provided strong fixation for a well-fixed implant not requiring revision to a longer-stemmed device [21].

A number of studies have shown that proximal screw configurations are stiffer than proximal cable systems, are equally as stiff as allograft constructs [1,17], and can provide higher load-to-failure resistance during heel strike [21]. Specifically, Dennis et al. compared the traditional Ogden construct using proximal cables and distal bicortical screws with a construct composed of a lateral and medial allograft strut fixed with cables [17]. In torsional load-to-failure, the Ogden system provided 27% more rotational resistance to failure than the allograft arrangement. Similarly, Fulkerson and co-investigators compared an Ogden construct with a locked plate system fixed with proximal unicortical and distal bicortical screws [18]. They discovered no statistically significant difference between these 2 systems in torsional load-to-failure levels. Moreover, Schmotzer and colleagues biomechanically tested 6 different surgical management techniques for a fracture at the tip of a total hip arthroplasty [21]. Femurs were oriented at 15 degrees of flexion and 7 degrees of adduction to simulate loading during heel strike. Loads were gradually increased until failure occurred. They concluded that the use of proximal unicortical screws provided the highest load-to-failure resistance for a well-fixed implant not requiring revision to a longer-stemmed device.

### Factors Influencing Mechanical Stiffness

It must be noted that mechanical stiffness, as described in the present study, should not be considered the sole or best criterion in determining the clinical success of fracture fixation procedures. Other important outcome measures reported previously by the current authors and others include the static load required to cause complete failure of the bone-implant construct, the dynamic load required to instigate significant incremental deformation of a bone-implant system during cyclic loading, and the motion of bone fragments at the fracture site [1,4,17-24,31,32]. The questions that should always be considered are what outcome measure is most appropriate to assess and what level is needed to achieve optimal fracture site union and load sharing between bone and implant immediately following surgery and longterm. At present, mechanical stiffness was the sole criterion to evaluate immediate post-operative stability of a fracture fixation system. Longterm mechanical behaviour would require additional outcome measures for evaluation.

### Limitations

Firstly, all tests were done using quasi-static loads far below physiologic levels. For future studies, it is highly recommended that a test regime more representative of real-life conditions be employed, namely, an applied hip joint force of at least 3 times body weight [33], a cyclic force regime [22,33], and/or load-to-failure tests [22,24]. Such force regimes can or will lead to loosening and catastrophic failure of the repair construct in a physiologic manner [17,18]. More specifically, future investigators should consider performing load-to-failure tests, which could provide further information about whether the use of screws in the vicinity of a total hip stem creates stress risers leading to refracture. Presently, such tests could not be performed because the same femurs were used for measuring stiffnesses of the cable-plate and screw-plate systems.

Secondly, femurs were stripped completely of all soft tissue. The additional support to the repair constructs that would be provided by surrounding soft tissues during physiological conditions was not considered. Thus, current stiffness results may underestimate the overall stiffnesses experienced in vivo.

Thirdly, by using the opposite intact femur as a control, this study did not separately and directly consider the influence of the prosthesis and cement on the stiffnesses measured. However, this was not the research question of interest at present. Moreover, because each specimen utilized an identical hip implant-cement configuration, any statistical differences were due to differences in shaft fracture repair technique.

Fourthly, the number of specimens for each construct group was limited (n = 4), likely yielding low statistical power for the investigation and leading to lack of detection of all actual statistical differences present, i.e. type II error. Previous investigators analyzing the biomechanics of periprosthetic B1 femoral fracture fixation have typically used 5 to 8 femurs per test group [1,17,18,22,24]. Conversely, however, the low number of specimens per group at present means that the several statistical differences detected were, in fact, present.

Fifthly, mechanical properties of the specimens were assumed to have been maintained over the duration of the study. However, a prior study on human femoral fracture fixation showed a 30% decrease in stiffness of repair constructs over several months during the testing period [34]. This may have been due to device migration/settling within the host bone, cumulative bone damage over time, and/or repeated thawing/freezing. The authors did not monitor these phenomena at present.

Sixthly, there are some clinical concerns regarding the way in which repair constructs were applied presently. Specifically, cortical screw tips could potentially breach the cement mantle, which could lead to substantial cement fracture and eventual hip implant loosening. Moreover, cortical screw tips could nick the lateral surface of the hip stem, thereby generating metallic wear debris during patient activities. In addition, some of the mechanical stiffness measured may have been due to screw impingement into cement, thus slightly overestimating the stiffness levels that could be achieved in vivo. Care should be taken in choosing the appropriate screw length, especially for insertion points in the proximal femur in the proximity of the hip stem. Consequently, the results of this investigation cannot be generalized for all Vancouver B1 fracture fixation constructs using screw-plate and cable-plate systems, but only for those fractures in the presence of hip implants that have been well fixed with cement.

Finally, present stiffnesses of bone-implant constructs may be different compared to clinical conditions. Testing was done with cortical contact between fracture fragments using an idealized oblique osteotomy with perfect matching between fracture segments, thereby enhancing inter-fragment surface friction. However, clinically-performed fracture reductions are never perfectly matching; they can yield lower or higher stiffnesses depending on the jaggedness of the fracture line and the success of inter-fragment matching. Different results may also be obtained with some comminution or gap at the fracture site, which may be a more problematic injury pattern clinically. Moreover, because several investigations in addition to the present study have examined the B1 fracture, future work should consider B2 and B3 fractures.

## Conclusions

In axial compression, all constructs demonstrated statistically significant improvement in biomechanical stiffness over intact femur baselines values. However, four-point bending and torsional stiffnesses yielded values that were lower than intact controls, except in a few instances. For a given construct, screw-plate systems were stiffer than cable-plate systems in about half of all cases assessed and were equally as stiff as cable-plate systems in the remaining situations. This suggests that when maximal stability is required for periprosthetic fracture fixation, a plating system using proximal and distal screw fixation is one option for the surgeon. With cortical contact, the type of plate used has only a limited influence on the stability of periprosthetic fracture fixation. Finally, unlike prior investigations, this study also directly compared 3 cable-plate systems in which the manner of cable capture by the plate was varied.

## Abbreviations

A: Zimmer Cable Ready System; B: AO Cable-Plate System; C: Dall-Miles Cable Grip System; ANOVA: analyses of variance; p: statistical difference criterion

## Competing interests

The authors declare that they have no competing interests.

## Authors' contributions

JPL was involved in initial study design, femur acquisition, implant acquisition, specimen preparation, specimen testing, and statistical analysis. RZ did the literature search, manuscript writing, figure preparation, and statistical analysis. MTN engaged in both specimen preparation and mechanical testing. JPW and EHS were involved in initial study design, implant acquisition, and general supervision of the project. All authors approve of this manuscript version.
